# Left Ventricular Global Longitudinal Strain Predicts Pacemaker-Associated Cardiomyopathy with Substantial LVEF Deterioration: Results from a Single-Center Cohort Study in Germany

**DOI:** 10.3390/jcm15062361

**Published:** 2026-03-19

**Authors:** Carlos Plappert, Philipp Lacour, Abdul S Parwani, Leif-Hendrik Boldt, Felix Bähr, Doreen Schöppenthau, Henryk Dreger, Emanuel Heil, Felix Hohendanner, Gerhard Hindricks, Jonas Lübcke, Ingo Hilgendorf, Florian Blaschke

**Affiliations:** 1 Department of Cardiology, Angiology and Intensive Care Medicine, German Heart Center at Charité, Campus Virchow Klinikum, 13353 Berlin, Germanyhenryk.dreger@dhzc-charite.de (H.D.);; 2 DZHK (German Centre for Cardiovascular Research), Partner Site Berlin, 10785 Berlin, Germany; 3 Kardiologie im Spreebogen and IB Hochschule Berlin, 12683 Berlin, Germany; 4 Department of Cardiology, Angiology and Intensive Care Medicine, German Heart Center at Charité, Campus Mitte, 10117 Berlin, Germany; 5 Department of Cardiology, Klinikum Brandenburg, 14770 Brandenburg, Germany

**Keywords:** right ventricular pacing, left ventricular ejection fraction, global longitudinal strain, heart failure, prognosis

## Abstract

**Background and Aims**: Permanent pacemaker (PM) implantation is an established treatment for symptomatic bradycardia. However, chronic right ventricular pacing (RVP) is associated with increased morbidity and mortality due to electrical and mechanical dyssynchrony, leading to pacing-induced cardiomyopathy (PICM). Prognostic markers for identifying patients at high risk of PICM remain scarce. This study compares patients with low (<30%) and high (≥30%) RVP burden with respect to echocardiographic parameters and clinical outcomes. **Methods**: This retrospective, double-blinded, single-center study included 105 patients who underwent dual-chamber PM implantation. RVP burden, left ventricular ejection fraction (LVEF), global longitudinal strain (LV-GLS), and all-cause mortality were assessed to evaluate the impact of RVP on LV function and clinical outcomes. **Results**: At baseline, the mean LVEF was 61 ± 6% and LV-GLS was 18 ± 4%. LVEF declined in seven patients (6.7%) during a mean follow-up of 30 ± 14 months, with a mean reduction from 56.1 ± 4.9% to 40.1 ± 5.0% (median 55% to 41%), thereby fulfilling the prespecified PICM definition (≥10% decrease from baseline >50%, excluding alternative causes). Of the 105 patients, 58 (55%) were classified into the low RVP group (<30%) and 47 (45%) into the high VP group (≥30%). High VP burden was associated with deterioration in both LVEF (6/47 [13%] vs. 1/58 [2%], *p* < 0.05) and LV-GLS (28/47 [60%] vs. 16/58 [28%], *p* < 0.001). In multivariable analysis, baseline LV-GLS was significantly associated with subsequent LVEF decline (OR 1.410, 95% CI 1.201–1.610, *p* < 0.001), and high VP burden was linked to LV-GLS decline (OR 1.358, 95% CI 1.160–1.534, *p* < 0.01). Kaplan–Meier analysis showed that time to LVEF deterioration (7 events) was significantly shorter in the high VP burden group (45.2 ± 2.9 vs. 55.7 ± 1.0 months, *p* < 0.05). Early LV-GLS decline within 1 year predicted subsequent LVEF deterioration (HR 7.210, 95% CI 4.239–9.516, *p* < 0.05), with a significantly shorter time to LVEF deterioration in these patients (34.7 ± 4.2 vs. 53.7 ± 1.4 months, *p* < 0.001). All-cause mortality did not differ significantly between high and low VP burden groups (*p* = 0.2). **Conclusions**: In patients with normal preimplant LVEF and ≥30% RVP, LV-GLS decline of >10% from baseline serves as an early and sensitive marker for subsequent LVEF deterioration and is associated with adverse outcomes. Early LV-GLS monitoring may help identify patients at higher risk for progressive ventricular dysfunction.

## 1. Introduction

Permanent pacemaker (PM) implantation in patients with cardiac conduction disorders has significantly reduced symptom burden, improving quality of life and patient-centered outcomes over recent decades [1,2]. Although these benefits are well-established and endorsed by recent guidelines from the European Society of Cardiology (ESC) and the European Heart Rhythm Association (EHRA) [1,2,3], there is growing concern about potential long-term adverse effects.

Pacing-induced cardiomyopathy (PICM) is a serious complication of right ventricular pacing (RVP) [4,5]. Pathophysiologically, RVP induces an abnormal activation pattern resembling left bundle branch block (LBBB), resulting in electromechanical dyssynchrony, prolonged QRS duration, and impaired myocardial efficiency [2,6]. RVP and the development of PICM are associated with substantial cardiovascular morbidity and mortality [6,7,8]. The detrimental effects of RVP, including an increased risk of heart failure (HF) with reduced left ventricular ejection fraction (LVEF) and HF-related hospitalizations, have been shown in large clinical trials [6,9,10]. The Mode Selection Trial in Sinus Node Dysfunction (MOST) trial, which randomized 2010 patients to dual- or single-chamber pacemakers, found that high RVP burden was associated with increased HF hospitalizations and new-onset atrial fibrillation (AF) [7]. Similarly, the Dual Chamber and VVI Implantable Defibrillator (DAVID) trial demonstrated that high VP burden was linked to higher mortality risk [8].

The incidence of PICM is influenced by several risk factors, including male sex, lower baseline LVEF, history of myocardial infarction (MI), AF, chronic kidney disease (CKD), wider native or paced QRS durations, and high RV pacing burden [5,11]. Biventricular cardiac resynchronization therapy (CRT) and conduction system pacing (CSP), including His-bundle pacing, have emerged as promising alternatives to reduce dyssynchrony. However, current guidelines do not yet recommend these strategies for patients with preserved LVEF [2].

Although RVP is often well-tolerated for many years without adverse effects, a minority of patients with initially normal LVEF, just over 10%, develop PICM [5,12]. However, identifying high-risk patients remains challenging, and robust prognostic markers are lacking. This underscores the need for improved risk stratification, as early identification of vulnerable patients is crucial. We aimed to identify prognostic factors for the development of PICM, stratified by VP burden.

## 2. Methods

### 2.1. Study Design

This retrospective, double-blinded, single-center study evaluated the impact of RVP on cardiac structure and function, focusing on echocardiographic parameters.

### 2.2. Study Participants

**Figure 1:**
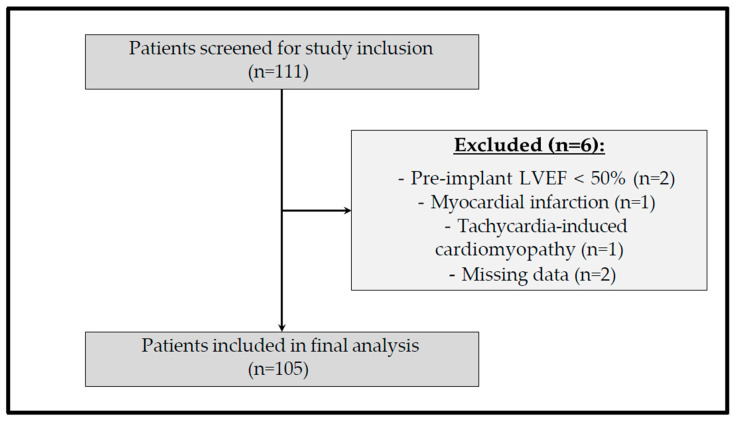
Flowchart illustrating screening, exclusions, and final inclusion of patients.

A total of 111 patients aged ≥ 18 years who underwent dual-chamber PM implantation at the Department of Cardiology, Charité Campus Virchow Klinikum, a tertiary care university hospital in Berlin, Germany, between April 2011 and July 2016 were enrolled. A baseline LVEF ≥ 50% was confirmed by transthoracic echocardiography within six months prior to implantation. Exclusion criteria comprised LVEF < 50%, missing follow-up echocardiographic data, inadequate image quality, or LVEF decline due to acute cardiac events (e.g., MI or tachycardia-induced cardiomyopathy). All patients provided written informed consent. The study was approved by the institutional ethics committee and conducted in accordance with the Declaration of Helsinki. The patient selection process, including screening, exclusions, and final cohort size, is illustrated in Figure 1.

### 2.3. Patient Data Collection, Follow-Up, and Clinical Outcomes

Data were collected from all eligible patients, including sociodemographic characteristics, medical history, comorbidities, vital signs, physical examination and symptom assessment (heart rate, body mass index, New York Heart Association [NYHA] class), as well as current medications, electrocardiogram (ECG), and echocardiography. Pacemaker manufacturer and lead positions were documented.

Follow-up with device interrogation was conducted at 6 weeks and annually thereafter at the cardiology outpatient clinic (Charité Campus Virchow Klinikum), assessing sensing and pacing parameters. Echocardiography followed a standardized protocol (Section 2.3.1). Mortality was ascertained via the Berlin Population Register at the end of follow-up.

The primary endpoint was the assessment of LVEF deterioration, with secondary endpoints including LV-Global longitudinal strain (GLS) decline and all-cause mortality during a mean follow-up of 30 ± 14 months.

#### 2.3.1. Echocardiographic Protocol and Analysis

Two-dimensional transthoracic echocardiography was performed using General Electric (GE) systems, in accordance with guidelines from the American Society of Echocardiography (ASE) and the European Association of Cardiovascular Imaging (EACVI) [13]. All examinations were conducted by an experienced echocardiographer blinded to pacing burden and clinical outcomes, with measurements averaged over three cardiac cycles. LVEF was calculated using the modified biplane Simpson’s method. LV-GLS was derived from apical two-, three-, and four-chamber views using a standard 18-segment model and analyzed using EchoPac software (version 110.1.0). To ensure reproducibility, image acquisition and speckle-tracking analysis followed a standardized protocol, and only high-quality loops were used for strain processing according to current ASE/EACVI recommendations.

#### 2.3.2. Electrophysiological Data Collection and Analysis

Electrocardiographic parameters were obtained from the 12-lead ECGs closest to pacemaker implantation (pre- and post-implantation). Parameters included heart rate, atrial excitation, atrioventricular (AV) conduction time, QRS duration (native or paced), and the presence of LBBB or RBBB. Ventricular pacing was documented or induced. Bundle branch blocks were defined according to standard criteria [14,15].

#### 2.3.3. Pacemaker Programming

Pacemaker systems from major manufacturers were implanted across the cohort, including Medtronic (Minneapolis, MN, USA), St. Jude Medical/Abbott (Abott Park, IL, USA), Biotronik (Berlin, Germany), and Boston Scientific (Marlborough, MA, USA).

Algorithms designed to minimize RVP, such as Managed Ventricular Pacing^TM^ (MVP) and Search AV+^TM^ (Medtronic), Ventricular Intrinsic Preference^TM^ (VIP) (St. Jude Medical/Abbott), AV-hysteresis (Biotronik), or RhythmIQ^TM^ (Boston Scientific), were available but not purposefully activated. PM programming otherwise followed standard institutional practice, and AV-delays were not intentionally prolonged to suppress RVP. Thus, RVP burden reflects intrinsic pacing requirements rather than device-driven pacing minimization.

#### 2.3.4. Definition of PICM and Device Interrogation Metrics

PICM was defined, according to current guidelines [16], as an absolute reduction of ≥10% percentage points in LVEF from a baseline >50%, with exclusion of alternative causes. LV-GLS deterioration was defined as a relative decline of >10% from baseline values. For example, an LVEF decrease from 58% to 48% or an LV-GLS change from −18% to −16% would meet these criteria. As no universally accepted GLS-based definition of PICM exists, the >10% relative LV-GLS decline was chosen in line with prior studies using GLS as an early marker of subclinical LV dysfunction and to exceed typical inter-observer variability in speckle-tracking measurements [17]. This threshold was intended to capture early functional impairment rather than to redefine PICM itself and is consistent with reports indicating that a relative GLS reduction of ≥10% represents a clinically meaningful change in longitudinal strain imaging.

Pacemaker interrogations included assessment of atrial and ventricular sensing and pacing percentages, as well as impedance, amplitude, sensing, and stimulation thresholds. Battery status was documented. Detected arrhythmias were reviewed by an experienced rhythmologist regarding therapeutic relevance. Ventricular pacing percentages were calculated per patient from device interrogation data. *VP*_1_ and *time*_1_ referred to pacing burden and duration from implantation to the first follow-up; *VP*_2_ and *time*_2_ to the interval from first to second follow-up, and so on as summarized in the following equation.

V P_total_ = V P_1_ · time_1_ + V P_2_ · time_2_ + … + V P_n−1_ · time_n−1_ + V P_n_ · time_n_

*time*_1_ + *time*_2_ + … + *time_n_*_−1_ + *time_n_*

### 2.4. Data Analysis and Statistical Methods

Continuous variables were presented as mean ± standard deviation (SD) or median (interquartile range [IQR]), and categorical data as counts and percentages. Receiver operating characteristic (ROC) analysis was performed, and the optimal VP cutoff was identified using the Youden index (J = sensitivity + specificity − 1), classifying patients into low and high VP burden groups. Group comparisons were performed using the Student *t*-test, Wilcoxon rank sum test, Chi-squared (χ^2^) test, Fisher’s exact test (when appropriate), or ANOVA. Univariable and multivariable logistic regression assessed associations between baseline characteristics and LVEF or LV-GLS deterioration. Models were adjusted for age, sex, and significant confounders (e.g., ischemic heart disease [IHD], MI). Univariable and multivariable effects were reported as regression coefficients, odds ratios (ORs), and 95% confidence intervals (CIs). Cox regression was reported as hazard ratios (HRs) and 95% CI. Kaplan–Meier curves for survival free from LVEF decline were generated according to LV-GLS deterioration. Censoring was applied to data from surviving patients at the last known follow-up to account for the lack of observed events. We tested our findings by comparing three VP burden categories (low: <5%, medium: 5–30%, high: 30–100%) using χ^2^ test, ANOVA, and Post Hoc analysis. Spearman correlation was used to assess echocardiographic variable correlations. Analyses were conducted using R statistical software (version 4.3.2). A two-tailed *p*-value < 0.05 was considered statistically significant.

## 3. Results

### 3.1. Baseline Characteristics

Of 111 screened patients for eligibility, 6 (5.4%) did not meet inclusion criteria or were excluded due to pre-implant LVEF < 50% (n = 2), MI (n = 1) or tachycardia-induced cardiomyopathy (n = 1), or missing data (n = 2). The final analysis included 105 patients (median [IQR] age 76 [69–80] years; 56 [53.3%] male; LVEF 60 [60–65]%; LV-GLS 18 [16–20]%).

**Figure 2:**
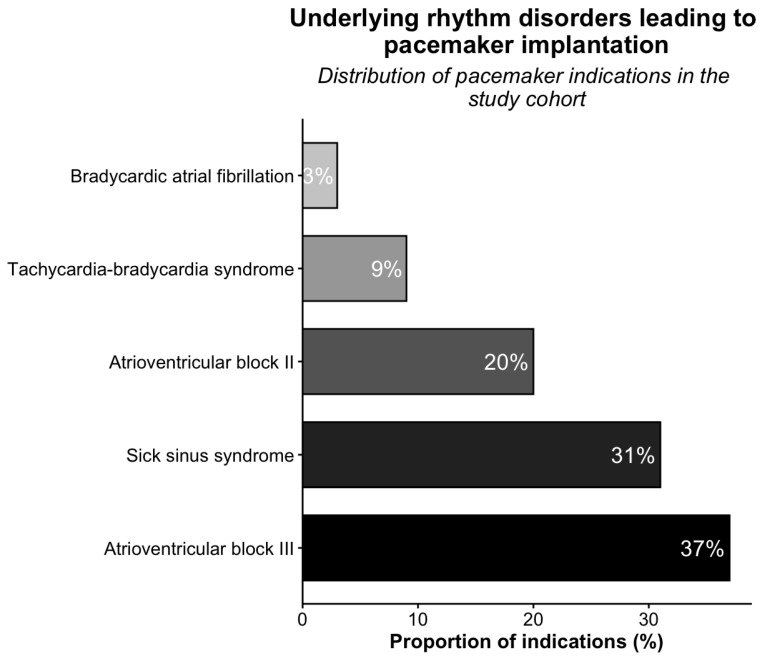
Distribution of pacemaker indications in the study cohort.

Baseline characteristics are summarized in Table 1. Hypertension (93.3%) and dyslipidemia (53.3%) were the most common comorbidities. Most patients were in NYHA class I (69.5%), and 76 (72.4%) had LV hypertrophy. The leading indications for PM implantation were AV block III (37%) and sick sinus syndrome (SSS) (31%) (Figure 2). Preimplant ECG showed LBBB in 23 patients (21.9%) and a mean QRS duration of 111 ± 27.2 ms.

Most patients received beta-blockers (68.3%) and angiotensin-converting enzyme (ACE) inhibitors (59.6%), followed by statins and diuretics. The mean follow-up duration was 29.6 ± 13.9 months. To provide additional descriptive insight, we performed a correlation analysis between LVEF and LV-GLS, revealing a strong positive association (r = 0.61, *p* < 0.001; Supplementary Figure S1).

### 3.2. Classification of Patients Based on Ventricular Pacing Burden

Receiver Operating Characteristics (ROC) analysis identified a 30% VP cutoff associated with significant deterioration in both LVEF and LV-GLS. Among the 105 patients, 58 (55.2%) were classified into the low VP (<30%) and 47 (44.8%) into the high VP (≥30%) groups. Sensitivity and specificity for LVEF deterioration were 0.86 and 0.58, respectively, with an Area Under the Curve (AUC) of 0.67. For LV-GLS deterioration, sensitivity was 0.64, specificity 0.69, and AUC 0.68.

The mean follow-up duration did not differ significantly between groups (29.7 ± 13.7 vs. 29.3 ± 14.2 months, *p* > 0.05). Characteristics of LVEF and LV-GLS deterioration by VP burden are shown in Supplementary Table S1.

### 3.3. Echocardiographic Assessment of LVEF and LV-GLS by Ventricular Pacing Burden Groups

**Figure 3:**
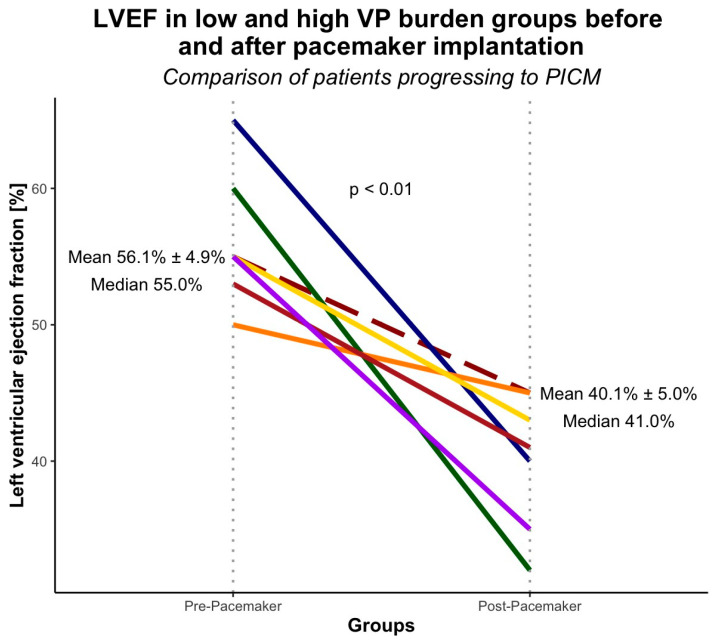
LVEF in patients progressing to PICM, stratified by VP burden groups at baseline (pre-pacemaker) and at last follow-up after pacemaker implantation. Each colored solid line represents an individual patient’s LVEF trajectory from baseline to follow-up in the high VP burden group, whereas the dashed line represents the mean LVEF trajectory of the low VP burden group. Different colors (blue, green, orange, purple, yellow, brown, etc.) are used solely to distinguish individual patients and do not represent specific subgroups. Vertical dotted lines indicate the two time points (pre- and post-pacemaker).

**Figure 4:**
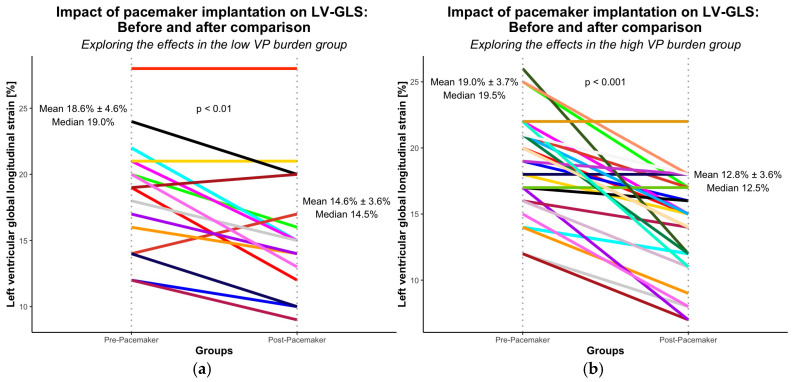
LV-GLS, stratified by VP burden groups at baseline and at last follow-up after pacemaker implantation: (**a**) low VP burden group and (**b**) high VP burden group. Each colored solid line represents an individual patient’s LV-GLS trajectory between baseline and follow-up. Different colors (blue, green, orange, purple, yellow, brown, etc.) are used solely to distinguish individual patients and do not represent specific subgroups. Vertical dotted lines indicate the two time points (pre-pacemaker and post-pacemaker). Mean and median LV-GLS values at each time point are displayed.

At the time of last echocardiographic follow-up (mean 40 ± 13 months), LVEF deterioration fulfilling PICM criteria was observed in 7 patients, predominantly in the high VP burden group (*n* = 6) compared to the low VP burden group (*n* = 1) (Figure 3). LVEF declined significantly from pre- to post-implantation (*p* < 0.01). LV-GLS decline occurred in both the low (*n* = 16) and high (*n* = 28) VP burden groups (Figure 4), with significant changes in each group (*p* < 0.01 and *p* < 0.001, respectively).

### 3.4. Impact of Ventricular Pacing on Echocardiographic Markers of Systolic Function

#### 3.4.1. Univariable Regression Analysis

Baseline characteristics stratified by LVEF and LV-GLS deterioration are shown in Table 2. Overall, 7 (6.7%) experienced LVEF decline and 44 (41.9%) showed LV-GLS deterioration.

**Figure 5:**
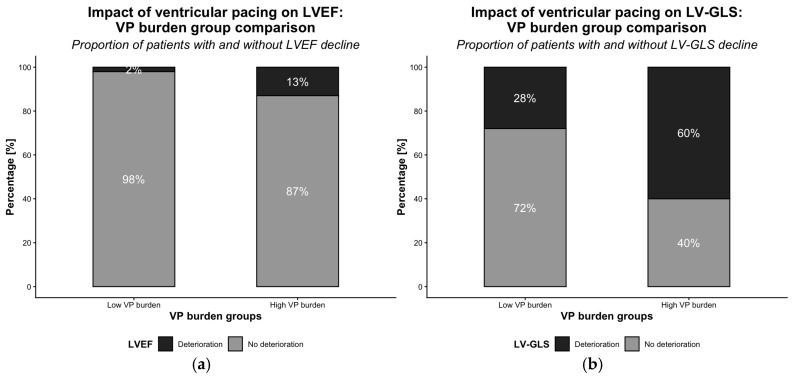
LVEF (**a**) and LV-GLS (**b**) deterioration stratified by VP burden groups.

LVEF decline was more common in the high VP group (13%, 6/47) than in the low VP group (2%, 1/58; *p* < 0.05) (Figure 5a). In univariable analysis, LVEF decline was significantly associated with high VP burden (*p* < 0.05), early LV-GLS deterioration (<1 year; *p* < 0.01), diabetes mellitus (*p* < 0.01), and LBBB (*p* < 0.05). LV-GLS deterioration occurred more frequently in patients with high VP burden (60%, 28/47) compared to those with low VP burden (28%, 16/58; *p* < 0.001) (Figure 5b). Univariable analysis identified high VP burden (*p* < 0.001), hyperlipidemia (*p* < 0.05), and LVH (*p* < 0.05) as significant correlates.

#### 3.4.2. Multivariable Logistic Regression Analysis

**Figure 6:**
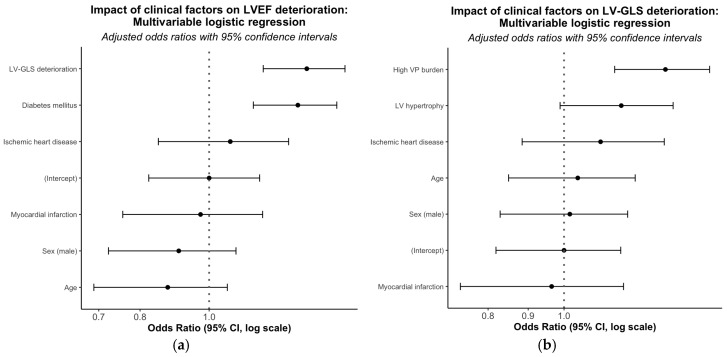
Forest plots of adjusted odds ratios (OR, 95% CI) for predictors of LVEF (**a**) and LV-GLS (**b**) deterioration. The vertical dashed line indicates the line of no effect (OR = 1). IHD, ischemic heart disease; MI, myocardial infarction; VP, ventricular pacing.

In the multivariable model adjusted for age, sex, and relevant confounders, early LV-GLS deterioration (OR 1.41, 95% CI 1.20–1.61; *p* < 0.001) and diabetes mellitus (OR 1.33, 95% CI 1.15–1.51; *p* < 0.01) were independently associated with LVEF decline. LV-GLS deterioration was independently associated with high VP burden (OR 1.36, 95% CI 1.16–1.53; *p* < 0.001) and LVH (OR 1.22, 95% CI 1.04–1.40, *p* < 0.05) (Table 3, Figure 6).

#### 3.4.3. Time-to-Event and Cox Regression Analysis of LV-GLS and LVEF Deterioration

In the overall cohort, the mean duration to LVEF and LV-GLS decline was 51 ± 1.7 months and 27 ± 2.4 months, respectively. Time to deterioration differed significantly between the high and low VP burden groups (45.2 ± 2.9 months vs. 54.7 ± 1.0 months for LVEF, *p* < 0.05; 20.5 ± 2.6 months vs. 33.8 ± 3.9 months for LV-GLS, *p* < 0.01).

In Cox regression analysis, LV-GLS decline within 1 year (HR 7.21, 95% CI 4.24–9.52, *p* < 0.05) was a significant predictor of subsequent LVEF deterioration. High VP burden (HR 2.04, 95% CI 1.53–4.21, *p* < 0.05) and LVH (HR 2.50, 95% CI 1.44–4.92, *p* < 0.05) were significant predictors of LV-GLS deterioration.

The number of patients with LV-GLS deterioration at 3, 12 and 36 months post-PM implantation was evaluated by VP burden groups. A significant difference was observed at 36 months (77% vs. 42%, *p* < 0.001), while no significant differences were found at 3 and 12 months (*p* = 0.29 and *p* = 0.31, respectively).

### 3.5. Stratified Internal Validation by VP Burden

**Figure 7:**
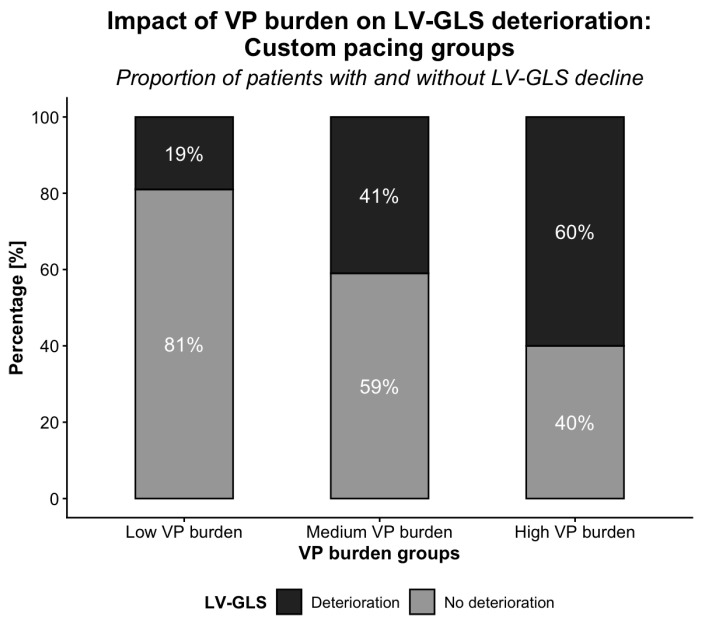
LV-GLS deterioration across predefined VP burden groups (<5%, 5–30%, ≥30%).

Patients were stratified into three predefined VP burden groups (low: <5%, medium: 5–30%, high: ≥30%) and assessed for LV-GLS deterioration (Figure 7).

LV-GLS deterioration occurred significantly more frequently in the high VP group than in both the medium (*p* < 0.01) and low (*p* < 0.001) groups. The difference between the low and medium groups was not statistically significant (*p* > 0.05). A log-rank test further confirmed a significant difference in LV-GLS deterioration between patients with VP < 5% vs. ≥5% (*p* < 0.01).

### 3.6. Impact of Early LV-GLS Decline on Subsequent LVEF Deterioration

**Figure 8:**
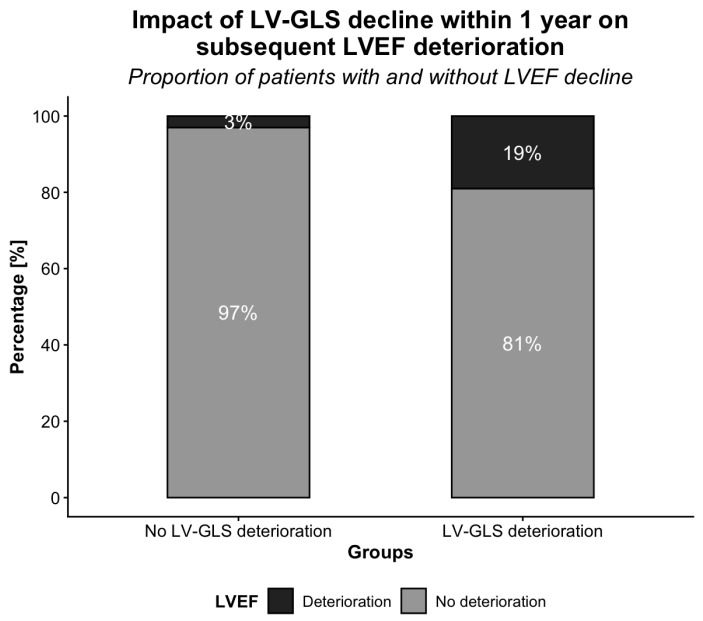
LVEF deterioration rates stratified by early LV-GLS decline within one year after pacemaker implantation.

**Figure 9:**
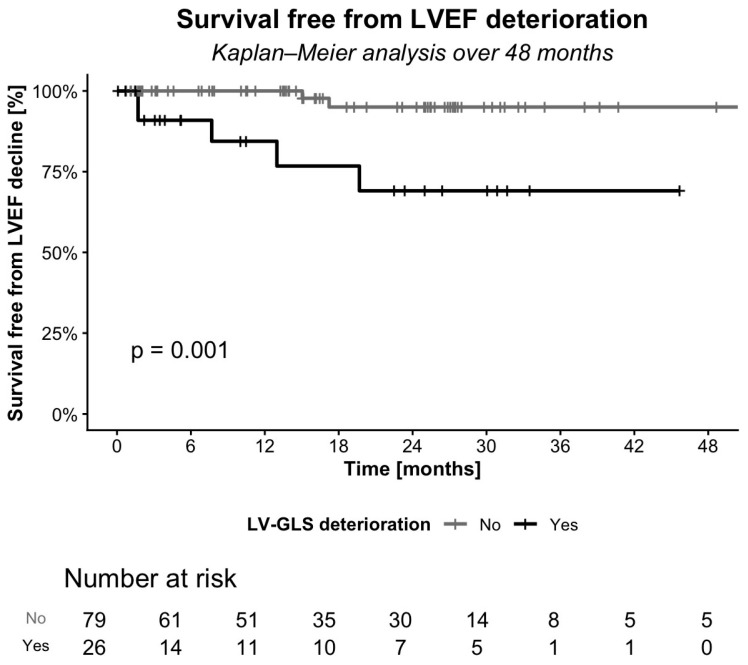
Kaplan–Meier curves showing survival free from LVEF decline in patients with and without early LV-GLS deterioration.

LV-GLS deterioration within 1 year after PM implantation was significantly associated with subsequent LVEF decline. Patients with early LV-GLS deterioration had a higher rate of LVEF decline (*p* < 0.001; Figure 8). The mean time to LVEF deterioration in the overall cohort was 51.0 ± 1.7 months. Among patients with early LV-GLS decline, time to LVEF deterioration was significantly shorter than in those without (34.7 ± 4.2 vs. 53.7 ± 1.4 months, respectively; *p* = 0.001; Figure 9). The Kaplan–Meier curves represent the estimated cumulative incidence of LVEF deterioration rather than the crude proportion. While the overall observed event rate was 7%, censoring and varying follow-up durations resulted in higher KM estimates (approximately 25% at 48 months). This is not inconsistent with the raw incidence but reflects the expected cumulative risk with longer follow-up.

### 3.7. Survival Analysis by VP Burden Groups

During follow-up, nine (8.6%) patients died in the study population. Mortality did not differ significantly between the low (three patients, 5.2%) and high (six patients, 12.8%) VP burden groups (*p* = 0.17). Similarly, a log-rank test showed no significant difference in mortality over time between the groups (*p* = 0.27).

## 4. Discussion

In patients undergoing RVP with preserved baseline LVEF, myocardial strain identified patients at risk for subsequent LVEF decline. The main findings were

1. Long-term LVEF decline occurred more frequently in patients with high VP burden (≥30%) compared with those with lower burden (<30%).
2. LV-GLS independently predicted subsequent LVEF deterioration in patients with preserved baseline LVEF and VP ≥30%, and was associated with adverse clinical outcomes.
3. Even moderate VP burden (>5%) was associated with measurable LV systolic impairment.

In our cohort, AV block II/III (57%) and SSS (31%) were the predominant indications for PM implantation, consistent with contemporary European and U.S. reports [18]. PICM is defined by a decline in LVEF accompanied by new-onset HF symptoms. Although no uniform definition exists, core criteria include preserved baseline LVEF, substantial RVP burden, and exclusion of alternative causes of LV dysfunction [11,16]. We defined PICM as baseline LVEF > 50%, an absolute LVEF reduction ≥ 10 percentage points, and exclusion of other cardiomyopathies, in accordance with these criteria [5].

The reported incidence of HF due to frequent VP ranges from 10% to 26%, with a commonly cited estimate of 12%. This variability reflects differences in PICM definitions, patient populations, and follow-up duration. In our cohort, the incidence was 6.7%, comparable to rates reported by Yu et al. (9%) and Ahmed et al. (7%) [5,11,19,20]. With an aging population and increasing PM implantation rates, PICM affects a growing patient population. However, only a subset of patients with high VP burden develops cardiomyopathy, and robust long-term predictors remain limited [5,11].

The RVP burden associated with adverse outcomes has been extensively investigated. In this study, an RVP burden ≥ 30% was defined as high and was associated with significant deterioration in LVEF and LV-GLS. Prior trials applied thresholds between 20% and 40%, linking higher RVP to new-onset HF, AF, and increased hospitalizations [7,8].

Observational studies demonstrate progressive LV systolic dysfunction with frequent RVP [19,21,22]. In our cohort, 85.7% of patients with substantial LVEF decline were in the high VP burden group. Khurshid et al. and Cho et al. reported LVEF reductions from 62.1% to 26.2% (mean follow-up 3.3 years) and from 60.5% to 40.1% (mean follow-up 4.7 years), respectively, for RVP ≥ 20% [21,22]. In our patients with RVP ≥ 30%, mean LVEF declined from 56.1% to 40.1% over 29.7 ± 13.7 months. The smaller absolute decline likely reflects shorter follow-up, given comparable PICM definitions across studies.

A VP threshold of 40% is commonly accepted; however, adverse effects have been reported at RVP ≥ 20% [21]. We therefore performed a stratified sub-analysis using predefined RVP groups: low (<5%, *n* = 36 [7 events]), medium (5–30%, *n* = 22 [9 events]), and high (30–100%, *n* = 47 [28 events]). Although subgroup sizes were modest, RVP > 5% was associated with LV systolic impairment and increased PICM risk, suggesting that adverse effects may occur below the conventional 40% threshold [21].

Several studies indicate that PICM is underestimated when defined solely by LVEF, as HF symptoms may occur despite preserved LVEF [18]. LV-GLS has emerged as a sensitive marker of early LV dysfunction and adverse remodeling [18,23], enabling detection of patients at risk for PICM before overt LVEF decline [20].

The clinical significance of a relative LV-GLS decline depends on baseline strain values. A 10% relative reduction from a normal baseline does not confer the same prognostic risk as an equivalent decline from an already impaired baseline. In our cohort, baseline LV-GLS independently predicted subsequent LVEF deterioration, indicating that both baseline strain severity and dynamic change inform risk stratification. Future studies should evaluate whether integrating relative and absolute LV-GLS thresholds improves prognostic discrimination. Consistent with this concept, recent data identified an absolute GLS threshold of approximately 16% associated with HF hospitalization despite preserved LVEF [24]. These findings support incorporating absolute strain thresholds alongside relative changes to improve risk stratification.

In multivariable analysis, early LV-GLS deterioration independently predicted subsequent LVEF decline, consistent with Xu et al., who likewise identified LV-GLS as an independent predictor of later LVEF deterioration (OR 1.41 vs. 1.62, respectively) [23]. Patients with impaired LV-GLS warrant close surveillance despite preserved LVEF.

Because LV-GLS deterioration within 12 months independently predicted subsequent LVEF decline in multivariable analyses, a pragmatic follow-up strategy includes baseline echocardiography with LV-GLS assessment prior to implantation, device interrogation at 6 weeks to quantify VP burden, and repeat echocardiography including LV-GLS at 12 months. A 6-month echocardiographic reassessment may be reserved for high-risk patients identified in our analyses, particularly those with high expected or documented VP burden (e.g., advanced AV block), LVH, DM, or impaired baseline LV-GLS. Beyond the first year, annual reassessment is appropriate in patients with persistently elevated VP burden or early strain abnormalities, reflecting the progressive LV-GLS decline observed during extended follow-up.

High RVP burden independently predicted LV-GLS decline. LV-GLS worsened after PM implantation in some patients with low VP burden, suggesting that PICM risk may begin below the commonly cited 20–40% threshold, warranting earlier clinical vigilance [7,8]. Although LV-GLS deterioration occurred more frequently than LVEF decline, this disparity reflects the greater sensitivity of strain for detecting subclinical dysfunction. Importantly, only patients with early LV-GLS decline progressed to subsequent LVEF deterioration, whereas those without strain decline largely preserved systolic function.

The Pacing and Ventricular Dysfunction (PAVD) trial showed that LV-GLS decline within 1 month after PM implantation predicted subsequent LVEF deterioration [20]. PICM; however, may develop between 1 month and 9 years post-implantation [22,25]. To our knowledge, this is the first study evaluating LV-GLS as a long-term marker of pacing-induced dysfunction. Time to pacing-induced LV dysfunction was shorter in patients with LV-GLS decline within 1 year than in those without (34.7 ± 4.2 vs. 53.7 ± 1.4 months). Follow-up duration exceeded that of prior studies (29.6 ± 13.9 vs. 12 months), and LV-GLS deterioration occurred later (7.6 ± 1.8 vs. 1 month) [20,23]. Differences in LV-GLS decline between VP burden groups emerged only after 36 months, not at 3 or 12 months, underscoring the importance of extended follow-up. LVH (HR 2.50) independently predicted LV-GLS deterioration, suggesting shared pathophysiological mechanisms such as prolonged intra-LV electromechanical delay.

Patients with pre-existing LVH are particularly vulnerable to the detrimental effects of chronic RVP. Hypertrophied myocardium is often accompanied by increased wall thickness, interstitial fibrosis, impaired microvascular perfusion, and reduced subendocardial reserve. Superimposed pacing-induced electrical dyssynchrony increases regional wall stress and exacerbates subendocardial dysfunction, accelerating adverse remodeling [18]. In this setting, even moderate dyssynchrony may precipitate early LV-GLS deterioration [26]. LVH thus represents a structural marker of reduced myocardial resilience to pacing-induced electromechanical delay [27].

Long-term outcomes in patients with PICM are poor [7,22]. New-onset AF is frequent and likely reflects structural and functional remodeling of the left atrium [18,28]. Pastore et al. reported a post-implantation AF incidence of 23.9%, comparable to our rate of 20% [29]. In a retrospective cohort of 1418 PM patients, Cho et al. observed an all-cause mortality of 11.3%, compared with 8.6% in our cohort. No significant difference in all-cause mortality was observed between VP burden groups. Only nine deaths occurred during follow-up, and the study was not powered for hard clinical endpoints. All patients had preserved baseline LVEF and were predominantly NYHA class I–II, constituting a low-risk cohort. The small number of events and limited follow-up preclude reliable mortality assessment. The absence of a mortality signal reflects limited statistical power rather than true equivalence.

### 4.1. Targeting Dyssynchrony: Future Strategies to Reduce PICM Risk

PICM may be prevented or reversed by correcting dyssynchrony. Beyond guideline- directed medical therapy, alternative pacing strategies comprise biventricular cardiac resynchronization therapy (BiV-CRT) and conduction system pacing (CSP). BiV-CRT improves LV function, quality of life, and clinical outcomes compared with RVP [11,18,19]. In the Pacing to Avoid Cardiac Enlargement (PACE) trial, LVEF remained stable with BiV-CRT but declined under RVP after 12 months [30]. CRT upgrade restores LVEF even in advanced dysfunction [31,32]. CSP (e.g., His-bundle pacing) narrows QRS duration and reduces HF symptoms and hospitalizations compared with RVP [11]. No clear superiority of BiV-CRT over CSP has been demonstrated [5,33]. Both strategies are limited by technical complexity and potential long-term complications [11,18,25].

Current guidelines do not recommend CRT or CSP in patients with preserved LVEF in the absence of HF symptoms [3,16], and randomized evidence in this population remain limited. Whether early CRT or CSP implantation in selected high-risk individuals improves outcomes sufficiently to justify procedural risks requires prospective evaluation [11,18].

Our findings support a risk-enriched trial design. Future randomized studies comparing CSP or CRT with conventional RVP should enroll patients with impaired baseline LV-GLS or early LV-GLS deterioration despite preserved LVEF, as these individuals represent a biologically vulnerable subgroup at increased risk of systolic dysfunction. In our cohort, baseline LV-GLS independently predicted LVEF decline, and early LV-GLS deterioration conferred a more than sevenfold increase in risk (HR 7.2), whereas VP burden alone did not consistently identify patients who developed PICM, indicating that myocardial strain rather than pacing percentage defines vulnerability. This concept aligns with mechanistic data identifying LV-GLS as an early marker of pacing-related myocardial susceptibility and with contemporary outcome studies demonstrating clinical benefit of physiologic pacing strategies [5,26,34]. A strain-guided enrichment strategy may improve statistical efficiency in prevention trials by increasing event rates.

To establish clinical utility, future multicenter trials should prioritize clinically meaningful endpoints over imaging surrogates. Prevention of LVEF decline represents a mechanistic outcome, whereas reduction in HF hospitalization reflects direct patient benefit. In populations with preserved LVEF, mortality alone is an impractical primary endpoint due to low event rates. Contemporary HFpEF and device trials therefore use composite endpoints, with treatment effects driven primarily by reductions in HF hospitalization and reverse remodeling rather than mortality alone [35,36]. A composite endpoint integrating LVEF deterioration, HF hospitalization, and device upgrade offers a pragmatic and clinically relevant design.

In patients with high VP burden and a >10% decline in LV-GLS at 12 months despite preserved LVEF and absence of symptoms, our data do not support immediate device upgrade. Subclinical strain deterioration should prompt intensified clinical and echocardiographic surveillance at 6-month intervals, including symptom assessment, repeat strain analysis, and reassessment of VP burden. Device interrogation should include AV delay optimization and activation of VP minimization algorithms to permit intrinsic conduction when feasible. Guideline-directed medical therapy (GDMT) should be optimized when additional CV risk factors are present.

Given that adverse effects were observed even at VP burdens > 5%, unnecessary right ventricular pacing should be minimized when feasible. Algorithms designed to promote intrinsic conduction and reduce cumulative VP exposure may mitigate pacing-induced dyssynchrony. However, excessive prolongation of AV delay may impair atrioventricular synchrony and adversely affect hemodynamics. Device programming should therefore be individualized to balance reduction in VP burden against preservation of physiological AV timing.

### 4.2. Study Limitations and Strengths

This single-center study included a limited number of patients, introducing potential selection and treatment bias. The small number of LVEF deterioration events (*n* = 7) limits statistical power and increases the risk of model overfitting; multivariable analyses for LVEF decline should therefore be considered exploratory. Covariate selection was restricted a priori to clinically relevant variables to mitigate low events-per-variable. The study was powered to detect differences in LV function between VP groups, but not for hard clinical endpoints. All-cause mortality was recorded; however, cause-specific mortality and systematically collected data on HF events or CRT upgrades were unavailable. We therefore cannot determine whether echocardiographic deterioration translated into symptomatic HF or device escalation. Some echocardiographic datasets were excluded due to inadequate image quality, potentially affecting measurement precision. Intra- and inter-observer variability for LVEF and LV-GLS were not formally assessed, which may reduce strain measurement precision, particularly in paced rhythms.

The 30% RVP threshold used to define high VP burden was derived within this cohort and lacks external validation. The association between VP burden and subsequent LV functional decline should therefore be interpreted cautiously, as internal derivation within a single cohort may overestimate predictive performance.

Patients with mildly reduced LVEF (41–49%) were excluded to ensure preserved systolic function at baseline and cohort homogeneity. The findings therefore cannot be generalized to patients with HFmrEF. Contemporary European registry data show higher mortality and HF hospitalization rates in patients with LVEF 41–49% compared with those with LVEF ≥ 50%, indicating that even modest systolic impairment carries adverse prognosis [37]. Reduced baseline systolic reserve may represent a more vulnerable myocardial substrate with increased susceptibility to pacing-induced dysfunction. The incremental prognostic value of LV-GLS-guided risk stratification in this population warrants evaluation in adequately powered retrospective studies.

The stratified analysis by VP burden should be interpreted with caution, as subgroup sizes were modest and the study was not powered for formal interaction testing. These findings are exploratory and hypothesis-generating.

## 5. Clinical Implications and Conclusions

Routine serial LV-GLS monitoring in all PM recipients is not warranted. Our data support a risk-adapted strategy. Patients with high VP burden (≥30%) exhibited the highest risk of LV-GLS decline and subsequent LVEF deterioration, although strain abnormalities also occurred at lower pacing percentages (>5%). Baseline and 12-month LV-GLS assessments are reasonable in most patients, while intensified surveillance should be reserved for individuals with high VP burden or additional vulnerability markers identified in our analyses. This selective approach enables early detection of pacing-induced dysfunction while preserving resources.

This study demonstrates that LV-GLS provides incremental prognostic information in patients with preserved pre-implant LVEF undergoing RVP. Early LV-GLS decline independently predicted subsequent LVEF deterioration and adverse outcomes, whereas pacing burden alone did not consistently identify vulnerable individuals. LV-GLS may refine risk stratification beyond pacing percentage. Prospective studies are required to confirm its clinical utility and determine whether strain-guided management improves outcomes.

## Figures and Tables

**Table 1:** Baseline characteristics of the study population.

Characteristics	Overall Study Population n = 105
Age, median (IQR), y	76 (69–80)
Sex, *n* (%)	
Male	56 (53.3)
Female	49 (46.7)
Heart rate, median (IQR), beats per minute	70 (69–70)
Body mass index, kg/m^2^	27 (24–30)
New York Heart Association group, *n* (%)	
Class I	73 (69.5)
Class II	25 (23.8)
Class III	6 (5.7)
Class IV	1 (1.0)
**Echocardiographic characteristics**	
Left ventricular ejection fraction, %	60 (60–65)
Left ventricular global longitudinal strain, %	18.0 ± 3.5
Left ventricular hypertrophy, *n* (%)	76 (72.4)
Right ventricular hypertrophy, *n* (%)	3 (2.9)
**Medical history,** ***n*** **(%)**	
Obesity (body mass index > 30 kg/m^2^)	23 (21.9)
Hypertension	98 (93.3)
Dyslipidemia	56 (53.3)
Diabetes mellitus	27 (25.7)
Smoking	27 (25.7)
Ischemic heart disease	48 (45.7)
Myocardial infarction	22 (21.0)
Atrial fibrillation	11 (10.5)
Chronic obstructive pulmonary disease	17 (15.9)
Pulmonary hypertension	33 (3.8)
Chronic kidney disease	46 (43.8)
Anemia	9 (8.4)
**Electrocardiogram characteristics**	
Left bundle branch block, *n* (%)	23 (21.9)
Right bundle branch block, *n* (%)	25 (23.8)
QRS, ms	105 (89–130)
**Medications,** ***n*** **(%)**	
Angiotensin-converting enzyme inhibitor	62 (59.6)
Angiotensin II receptor blocker	32 (30.8)
Beta-blocker	71 (68.3)
Calcium channel blocker	35 (33.7)
Loop diuretics	48 (46.2)
Statins	55 (52.9)

Continuous variables are presented as mean ± standard deviation or median (interquartile range) and categorical variables as *n* (%); *n*, number.

**Table 2:** Study characteristics associated with LVEF and LV-GLS decline.

Characteristics	No LVEF Decline	LVEF Decline	*p*-Value	No LV-GLS Decline	LV-GLS Decline	*p*-Value
Number	98 (93.3)	7 (6.7)		61 (58.1)	44 (41.9)	
Sex (male)	53 (54.1)	3 (42.9)	0.56	31 (50.8)	25 (56.8)	0.54
High VP burden	41 (41.8)	6 (85.7)	<0.05	16 (27.6)	28 (63.6)	<0.001
LV-GLS decline < 1 year	21 (21.4)	5 (71.4)	<0.01	-	-	-
**Medical history**						
Hypertension	92 (93.9)	6 (85.7)	0.40	55 (90.2)	43 (97.7)	0.13
Hyperlipidemia	53 (54.1)	3 (42.9)	0.60	38 (62.3)	18 (40.9)	<0.05
Diabetes mellitus	22 (22.4)	5 (71.4)	<0.01	15 (24.6)	12 (27.3)	0.76
Smoking	26 (26.5)	1 (14.3)	0.47	20 (32.8)	7 (15.9)	0.05
Ischemic heart disease	45 (45.9)	3 (42.9)	0.88	24 (39.3)	24 (54.5)	0.12
Myocardial infarction	21 (21.4)	1 (14.3)	0.65	12 (19.7)	10 (22.7)	0.70
Left ventricular hypertrophy	70 (71.4)	6 (85.7)	0.41	39 (63.9)	37 (84.1)	<0.05
Right ventricular hypertrophy	3 (3.1)	0	-	1 (1.6)	2 (4.4)	0.38
Pulmonary hypertension	29 (29.6)	2 (28.6)	0.95	14 (23.0)	17 (38.6)	0.08
Hypertensive heart disease	3 (3.1)	0	-	2 (3.3)	1 (2.3)	0.76
Peripheral vascular artery disease	15 (15.3)	1 (14.3)	0.94	7 (11.5)	9 (20.5)	0.21
Chronic obstructive pulmonary disease	16 (16.3)	1 (14.3)	0.89	12 (19.7)	5 (11.4)	0.25
Chronic kidney disease	43 (43.9)	3 (42.9)	0.96	27 (44.3)	19 (43.2)	0.91
**ECG parameters**						
Left bundle branch block	19 (19.4)	4 (57.1)	<0.05	14 (23.0)	9 (20.5)	0.76
Right bundle branch block	25 (25.5)	0	-	15 (24.6)	10 (22.7)	0.88

Results are presented as *n* (%) for categorical variables; VP, ventricular pacing; ECG, electrocardiogram.

**Table 3:** Multivariable logistic regression for predictors of LVEF and LV-GLS deterioration.

Characteristics	Estimate	Adjusted OR (95% CI)	*p*-Value
**LVEF deterioration**			
Age (per year)	−1.34	0.88 (0.83, 1.21)	0.16
Male (ref.)		1.00	
Female	−9.81	0.91 (0.72, 1.09)	0.30
LV-GLS deterioration (<1 year)	3.21	1.41 (1.20, 1.61)	<0.001
No history of diabetes mellitus (ref.)		1.00	
Diabetes mellitus	2.90	1.33 (1.15, 1.51)	<0.01
No history of ischemic heart disease (ref.)		1.00	
Ischemic heart disease	6.83	1.07 (0.85, 1.30)	0.55
No history of myocardial infarction (ref.)		1.00	
Myocardial infarction	−2.78	0.97 (0.76, 1.20)	0.80
**LV-GLS deterioration**			
Age (per year)	4.03	1.04 (0.85, 1.23)	0.68
Male (ref.)		1.00	
Female	1.70	1.02 (0.83, 1.21)	0.90
Low VP burden (ref.)		1.00	
High VP burden	3.10	1.36 (1.16, 1.53)	<0.001
No history of left ventricular hypertrophy (ref.)		1.00	
Left ventricular hypertrophy	1.68	1.22 (1.04, 1.40)	<0.05
No history of ischemic heart disease (ref.)		1.00	
Ischemic heart disease	1.07	1.11 (0.88, 1.34)	0.36
No history of myocardial infarction (ref.)		1.00	
Myocardial infarction	−3.65	0.97 (0.74, 1.19)	0.75

## Data Availability

All data generated or analyzed during this study are included in this published article. Further inquiries can be directed to the corresponding author.

## Supplementary Materials

The following supporting information can be downloaded at: https://www.mdpi.com/article/10.3390/jcm15062361/s1. Supplementary Figure S1: Scatter plot illustrating the correlation between LVEF and LV-GLS at baseline; Supplementary Table S1: Characteristics of LVEF and LV-GLS deterioration by ventricular pacing burden groups. LV, left ventricular; *n*, number.
