# Supplementary material for: Left Ventricular Global Longitudinal Strain Predicts Pacemaker-Associated Cardiomyopathy with Substantial LVEF Deterioration: Results from a Single-Center Cohort Study in Germany
Source: J Clin Med. 2026 Mar 19;15(6):2361. doi: 10.3390/jcm15062361 (PMC13026969; doi:10.3390/jcm15062361)
Supplement: Supplementary file 1 [file jcm-15-02361-s001.zip › jcm-4139740-supplementary.pdf]

SUPPLEMENTARY MATERIAL

SUPPLEMENTARY FIGURE

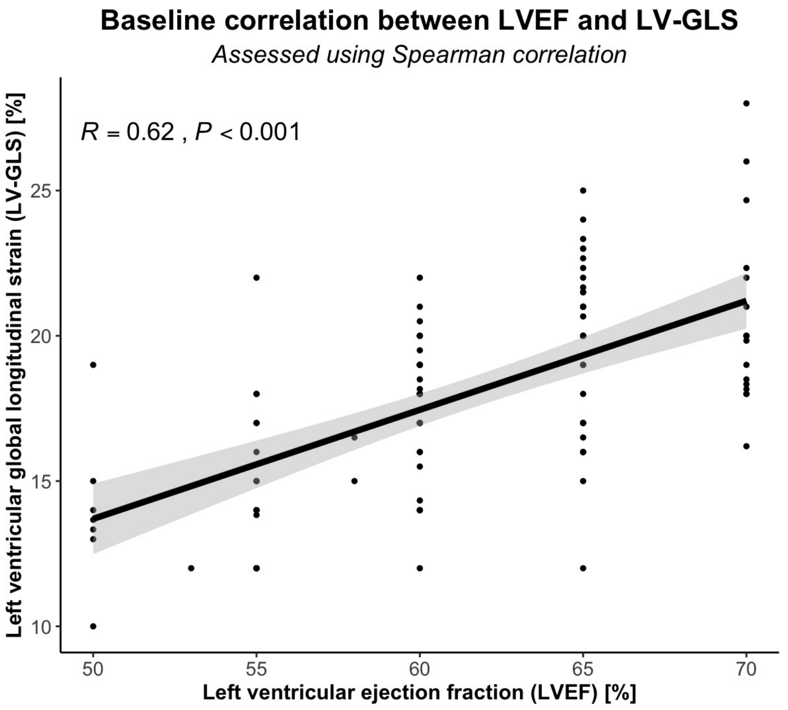

Supplementary Figure S1: Scatter plot illustrating the correlation between LVEF and LV-GLS at baseline.

SUPPLEMENTARY TABLE

Characteristics	Low VP burden group	High VP burden group
**LVEF deterioration**	(n = 1)	(n = 6)
Age, median (IQR), y	74.7	69.6 ± 7.2
Sex (male), *n* (%)	1	2 (33.3)
Left ventricular ejection fraction (preimplant)	55	56.7 ± 5.2
Left ventricular ejection fraction (postimplant)	45	40.0 ± 5.4
QRS duration (native)	112	140.4 ± 35.5
QRS duration (paced)	158	143.6 ± 30.8
Preimplantation, n (%)		
Atrioventricular Block	1	4 (66.7)
Sick sinus syndrome	-	2 (33.3)
Left bundle branch block	-	4 (66.7)
Right bundle branch block	-	
Left ventricular hypertrophy	-	5 (83.3)
Diabetes mellitus	1	4 (66.7)
Ischemic heart disease	1	2 (33.3)
Myocardial Infarction	1	-
**LV-GLS deterioration**	(n = 16)	(n = 28)
Age, median (IQR), y	75.6 ± 8.2	75.4 ± 7.8
Sex (male), *n* (%)	11 (68.8)	14 (50.0)
LV-global longitudinal strain (preimplant)	18.6 ± 4.4	19 ± 3.7
LV-global longitudinal strain (postimplant)	14.6 ± 3.6	12.8 ± 3.6
QRS duration (native)	90 (84-124)	119.6 ± 26.6
QRS duration (paced)	94 (86-158)	138.4 ± 30.2
Preimplantation, n (%)		
Atrioventricular Block	7 (43.8)	20 (71.4)
Sick sinus syndrome	2 (12.5)	5 (17.9)
Left bundle branch block	3 (18.8)	6 (21.4)
Right bundle branch block	3 (18.8)	7 (25.0)
Left ventricular hypertrophy	13 (81.3)	24 (85.7)
Diabetes mellitus	2 (12.5)	10 (35.7)
Ischemic heart disease	10 (62.5)	14 (50.0)
Myocardial Infarction	5 (31.5)	5 (17.9)

Supplementary Table S1: Characteristics of LVEF and LV-GLS deterioration by ventricular pacing burden groups. LV, left ventricular; n, number.
